# NCK-associated protein 1 regulates metastasis and is a novel prognostic marker for colorectal cancer

**DOI:** 10.1038/s41420-023-01303-6

**Published:** 2023-01-13

**Authors:** Mi Ri Kwon, Jae Hee Lee, Jin Park, Seok Soon Park, Eun Jin Ju, Eun Jung Ko, Seol Hwa Shin, Ga Won Son, Hye Won Lee, Yeon Joo Kim, Si Yeol Song, Seong-Yun Jeong, Eun Kyung Choi

**Affiliations:** 1 Department of Medical Science, Asan Medical Institute of Convergence Science and Technology, Asan Medical Center, University of Ulsan College of Medicine, Seoul, 05505 Republic of Korea; 2 Asan Institute for Life Sciences, ASAN Medical Center, Seoul, 05505 Republic of Korea; 3 Asan Preclinical Evaluation Center for Cancer Therapeutix, ASAN Medical Center, Seoul, 05505 Republic of Korea; 4 Department of Radiation Oncology, ASAN Medical Center, University of Ulsan College of Medicine, Seoul, 05505 Republic of Korea; 5 Department of Convergence Medicine, ASAN Medical Center, University of Ulsan College of Medicine, Seoul, 05505 Republic of Korea; 6 Present Address: Quality Evaluation Team, Samsung Bioepis Co., Ltd, 107, Cheomdan-daero, Yeonsu-gu, Incheon, 21987 Republic of Korea

**Keywords:** Tumour biomarkers, Metastasis

## Abstract

Metastatic colorectal cancer (CRC) remains a substantial problem for mortality and requires screening and early detection efforts to increase survival. Epithelial-mesenchymal transition (EMT) and circulation of tumor cells in the blood play important roles in metastasis. To identify a novel target for metastasis of CRC, we conducted a gene microarray analysis using extracted RNA from the blood of preclinical models. We found that NCK-associated protein 1 (NCKAP1) was significantly increased in the blood RNA of patient-derived xenograft (PDX) models of colon cancer. In the NCKAP1 gene knockdown-induced human colon cancer cell lines HCT116 and HT29, there was a reduced wound healing area and significant inhibition of migration and invasion. As the result of marker screening for cytoskeleton and cellular interactions, CRC treated with siRNA of NCKAP1 exhibited significant induction of CDH1 and phalloidin expression, which indicates enhanced adherent cell junctions and cytoskeleton. In HCT116 cells with a mesenchymal state induced by TGFβ1, metastasis was inhibited by NCKAP1 gene knockdown through the inhibition of migration, and there was increased CTNNB1 expression and decreased FN expression. We established metastasis models for colon cancer to liver transition by intrasplenic injection shRNA of NCKAP1-transfected HCT116 cells or by implanting tumor tissue generated with the cells on cecal pouch. In metastasis xenograft models, tumor growth and liver metastasis were markedly reduced. Taken together, these data demonstrate that NCKAP1 is a novel gene regulating EMT that can contribute to developing a diagnostic marker for the progression of metastasis and new therapeutics for metastatic CRC treatment.

## Introduction

Colorectal cancer (CRC) is the most common cancer in worldwide that ranked second for cancer mortality [1]. Despite progression in treatments, including surgical resection, radiation therapy, chemotherapy and targeted therapy, CRC remains a fatal disease with a high mortality of which 50% diagnosed, metastasized, resulting in a poor prognosis resulting in a poor prognosis [2]. The liver and lungs are the most common sites of metastasis from CRC [3–5].

Metastasis is a process by cancer cells disseminate into the vasculature from a primary tumor through epithelial-mesenchymal transition (EMT), circulate in the bloodstream, and extravasate into other organs [6]. The process of EMT involves cancer cells losing their epithelial characteristics, including cell adhesion, cell tight junctions, strong cytoskeleton structures, and E-cadherin (CDH1) and β-catenin (CTNNB1) expression levels followed by an increase in mesenchymal characteristics (fibroblast-like shape, upregulation of fibronectin (FN) and N-cadherin (CDH2)) [7]. In particular, transforming growth factor β1 (TGFβ1) is well known to be involved in EMT. TGFβ1-mediated EMT involves the suppressor of mothers against decapentaplegic (SMAD) or non-SMAD pathway. Furthermore, the non-SMAD pathway is associated with cell division control protein 42 homolog (CDC42)/Ras-related C3 botulinum toxin substrate 1 (Rac1) signaling for cell tight junctions [8].

Since early detection of metastatic cancer has been absolutely emphasized for treatment and prognosis, innovative diagnostic methods are being studied [9–14], such as detecting cancer-specific markers through liquid biopsy besides the common diagnosis, including imaging examinations and histopathologic examinations. A liquid biopsy is a biological fluid sample that can be obtained from the body, such as blood, saliva, urine, or spinal fluid [15]. It is easy to noninvasively obtain samples for liquid biopsy and provide real-time monitoring during the treatment period. Among liquid biopsy samples, blood contains considerable genetic and proteomic information related to tumors, such as circulating tumor cells (CTCs) and circulating tumor DNA (ctDNA). A CTC is a cancer cell that has detached from a solid tumor lesion and entered the peripheral blood circulation [16].

The CELLSEARCH® System is the first case that has been approved for clinical use by the Food and Drug Administration (FDA) and enables the detection and follow-up of cancer cells in the blood by observing the expression of epithelial cell adhesion molecule (EpCAM) and cytokeratin, which are known as representative cancer cell-specific molecular targets [17]. However, recent studies have shown that tumor cells acquire invasiveness in the early stage of metastasis. When they were introduced into blood vessels and circulated, their epithelial cell characteristics decreased, and mesenchymal cell characteristics were acquired, resulting in decreased expression levels of EpCAM and stem cell-like characteristics [18–21]. Therefore, discovering new metastatic cancer cell markers in the blood to replace EpCAM is necessary. For clinical applications, it is urgent to establish an in vivo or in vitro model for research on blood cancer cells.

Due to alterations in the primary tumor’s characteristics, some preclinical experiments do not match the results of clinical applications [22]. To overcome this disadvantage, we used patient-derived xenograft (PDX) models, which has the advantage of maintaining the originality of patient tumor samples.

In this study, we extracted RNA in blood from PDX models of pancreas, liver, lung, and colon cancer, which are well-known metastatic cancers, and conducted a microarray analysis to discover metastasis-specific CTC markers. We identified NCK-associated protein 1 (NCKAP1) as a novel biomarker for diagnosing CRC and predicting metastasis. NCKAP1 is known to interact with RAC1 and regulate the cytoskeleton [23, 24]. Currently, NCKAP1 is reported to be involved in poor prognosis and metastasis in hepatocellular carcinoma (HCC), breast cancer, and non-small-cell lung cancer (NSCLC) [25–29]. Although it has been reported that exosomes from colon cancer induce cancer invasion by regulating fibroblast focal adhesion protein and its actin cytoskeleton regulators, including NCKAP1 [30], NCKAP1 as a blood diagnostic marker in CRC is not yet known, and its metastatic mechanism has not been identified. Therefore, we evaluated the efficacy of NCKAP1 as a novel target in CRC and investigated the mechanism of metastasis inhibition of NCKAP1 through this study.

## Results

### Global gene expression profiling in the blood cells derived from PDX mice

**Fig. 1:**
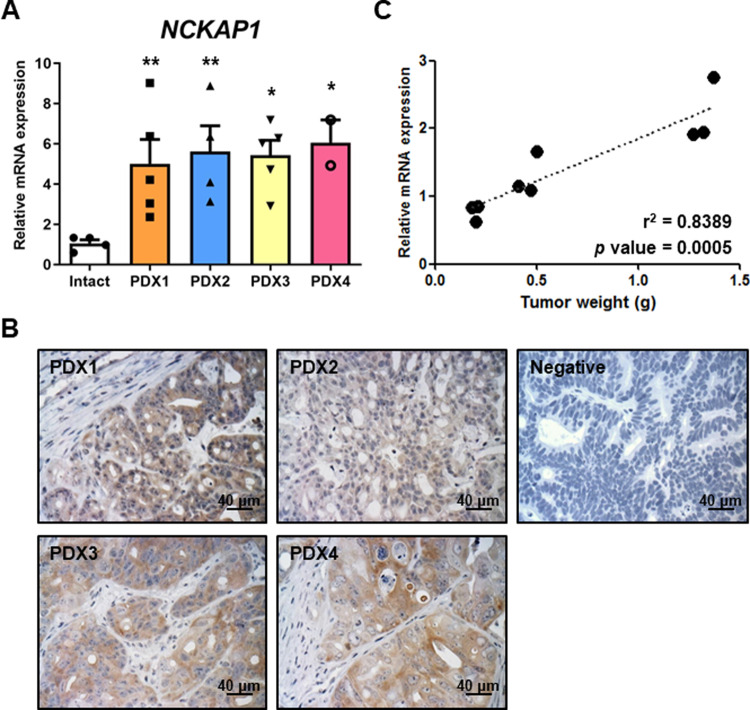
Correlation of NCKAP1 expression level and CRC. **A** The mRNA levels of NCKAP1 were confirmed by qRT-PCR analysis in blood cells derived from four colon cancer PDX models. Data are reported as the mean ± SEM. Student’s t-test *p < 0.05, **p < 0.01 vs. Intact. (Intact: *n* = 4, PDX1*: n* = 5, PDX2: *n* = 4, PDX3: *n* = 5, PDX4: *n* = 2). **B** IHC staining was performed with anti-NCKAP1. Bar, 40 μm. **C** Linear regression analysis was subsequently performed between relative mRNA levels of NCKAP1 and tumor weights. Data are reported as the mean ± SEM. Student’s t-test. *p* = 0.0005. (*n* = 9).

To identify a novel target of colon cancer cells and investigate its role in metastasis, we conducted gene microarray analysis using a liquid biopsy from preclinical models. The initial analysis was to select cancer types that were well-detected in blood. For the analysis, we screened epithelial marker expression levels in blood cells derived from various PDX models by quantitative PCR analysis for EPCAM and MUC1 genes. EPCAM and MUC1 are well-known epithelial cell markers used for the detection of CTCs. In the blood cells, we observed positive expression of EPCAM or MUC1 in lung and colon PDX models, and their expression levels were calculated by 2-dCt value for 1 μg of RNA (Fig. S1A). Colon cancer PDX was the only case in which EPCAM expression was positive in the blood. In this case, RNAs were pooled from three mice and subsequently used for microarray analysis. Among a total of 24,695 genes in the Affymetrix GeneChip® Human 2.0 ST array, 614 differentially expressed genes (DEGs) were identified by a two-fold cut-off. “Comparison 1” was selected as genes with high expression in the blood of tumor-bearing mice compared to normal blood without tumor. To metastasize, circulating tumor cells (CTCs) released from the primary tumor migrate through the blood. In this context what we focused on was that the fold change value would be different whether it was positive or negative when compared with the PDX tumor and PDX blood. To confirm this hypothesis, “Comparison 2” was performed. Up- and downregulated genes that were differentially expressed between intact and PDX mice are summarized in the schematic diagram and table (Fig. S1B). We selected several genes among DEGs to validate a microarray data, in which TRIM51, NCKAP1, MED27, and MCTP1 were included. As a result of the mRNA levels in the blood from two lung cancer PDX cases and two colon cancer PDX cases, significance was found only for NCKAP1 and MCTP1 (Fig. S1C and Fig. 1A).

### NCKAP1 as a novel target for CRC cells

We validated NCKAP1 expression in mRNA and protein levels using PDX models, and the relative values were calculated by comparing to intact mice. In the blood cells of PDX mice, a significant increase in NCKAP1 was confirmed by quantitative PCR analysis in four cases of colon adenocarcinoma (Fig. 1A). To investigate whether NCKAP1 can be applied as a diagnostic marker of colon cancer, whole blood samples were collected from patient-derived tumor-bearing mice at various time points, when tumor volumes reached at 250, 500, and 1000 mm^3^. Linear regression was subsequently performed between the relative mRNA levels of NCKAP1 and tumor weights, which were significantly correlated (Fig. 1C). NCKAP1 was also expressed in tumor mass as well as in the blood cells of colon cancer PDX mice. The expression was exhibited in the cytoplasm of epithelial and adenocarcinoma cells (Fig. 1B). These data suggested that NCKAP1 might be used as a novel target of colon cancer and applicable for diagnosis and monitoring via liquid biopsy.

### EMT blockade by reduction of NCKAP1 expression

**Fig. 2:**
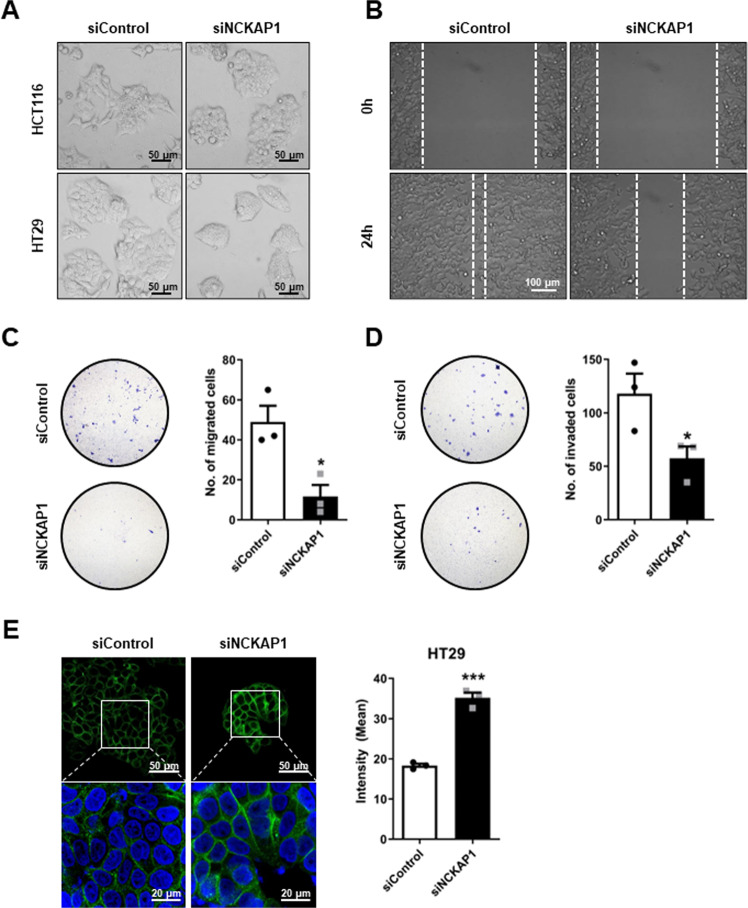
Inhibition of metastasis characteristics by reduction of NCKAP1. **A** HCT116-Luc and HT29-Luc cells were transfected with 20 nM siControl or 20 nM siNCKAP1 for 72 h. Transfected cells were observed under a phase-contrast microscope. Bar, 50 μm. **B** A wound healing assay was performed. HCT116-Luc cells were transfected with 20 nM siControl or 20 nM siNCKAP1 for 24 h and further seeded on Culture-Insert 2 Well for 24 h. After scratching, cell migration was monitored with a phase-contrast microscope for 24 h. Bar, 100 μm. **C, D** A migration and invasion assay of HCT116-Luc cells transfected with 20 nM siControl or 20 nM siNCKAP1 for 72 h was performed. **C** The migrated cells were stained with crystal violet and observed using a fluorescence microscope (10 X). Quantitative results on the right: Data are reported as the mean ± SEM. Student’s t-test. *p < 0.05 vs. siControl. (*n* = 3). **D** The invaded cells were stained with crystal violet and counted. Quantitative results on the right: Data are reported as the mean ± SEM. Student’s t-test. *p < 0.05 vs. siControl. (*n* = 3). **E** Transfected HT-29-Luc cells were subjected to immunocytochemistry of CDH1 and DAPI. Representative pictures of cells are shown by confocal microscope. Bar, 20 μm and 50 μm. Quantitative results on the right: Data are reported as the mean ± SEM. Student’s t-test. ***p < 0.001 vs. siControl. (n = 3).

To identify the function of NCKAP1 in metastasis in colon cancer, we conducted in vitro assays to test EMT ability. NCKAP1 was successfully knocked down by siRNA transfection in HCT116 and HT29 colon cancer cells, and its expression was confirmed by quantitative PCR and western blot (Fig. S3). The morphology was changed to a compact and round shape in siNCKAP1-treated cells (Fig. 2A). In EMT assays, siNCKAP1 treatment resulted in a decrease in the wound healing area for 24 h (Fig. 2B). Migration and invasion abilities were also inhibited by gene silencing of NCKAP1, and the differences in the number of cells passing through a Trans-well membrane were significant (Fig. 2C-D). CDH1 is one of the adherent molecules of cellular junctions and a major marker of epithelial cells. In siNCKAP1-treated colon cancer cells, the intensity of CDH1 expression was significantly increased (Fig. 2E). CTNNB1, another adherent protein, was also enhanced, as was CDH1 (Fig. S4A). NCKAP1 is a known member of a complex in the Rac pathway that regulates actin remodeling [31]. As actin has an important role in cell migration and cancer metastasis [32, 33], we analyzed the change in actin expression by NCKAP1. The actin cortex became thicker and stronger than control cells on confocal 3D images (Fig. S4B).

**Fig. 3:**
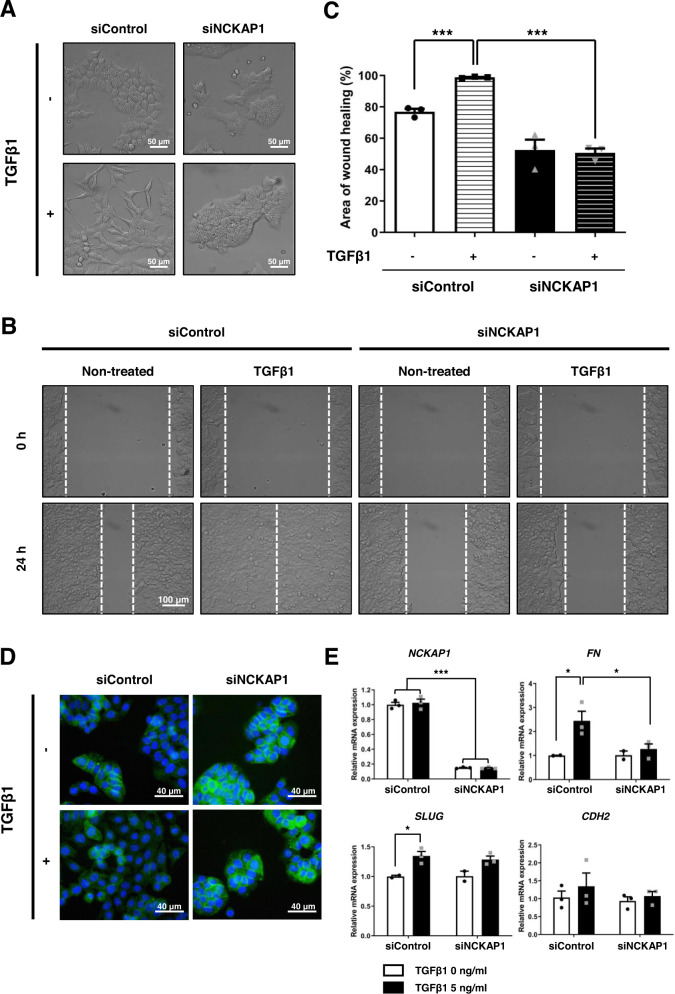
Inhibition of TGFβ1-induced EMT by reduction of NCKAP1. **A** HCT116-Luc cells were transfected with 20 nM siControl or 20 nM siNCKAP1 for two days and further treated with 5 ng/ml TGFβ1 for two days. Phase-contrast microscopy was performed. Bar, 50 μm. **B** Transfected HCT116-Luc cells were further treated with 5 ng/ml TGFβ1 for 24 h. After scratching, cell migration was monitored at the indicated time points and observed under a phase-contrast microscope. Bar, 100 μm. **C** The graphs quantitatively show the area of wound recovery in **B**. Data are reported as the mean ± SEM. One-way ANOVA and Tukey’s post hoc test. ***p < 0.0001 vs. TGFβ1 untreated siControl or TGFβ1 treated. (*n* = 3). **D** HT-29-Luc Cells were transfected with 20 nM siControl or 20 nM siNCKAP1 and further induced EMT with 5 ng/ml TGFβ1. Immunocytochemistry of CTNNB1 and DAPI was performed. Cells were observed under a confocal microscope. Bar, 40 μm. **E** The expression levels of mesenchymal related mRNA on HCT116-Luc cells were measured by qRT-PCR. Data are reported as the mean ± SEM. Two-way ANOVA and Bonferroni’s *post hoc* test. ***p < 0.0001 vs. siControl, *p < 0.05 vs. untreated TGFβ1 or siControl. (*n* = 3).

TGFβ1 has many cellular functions, including proliferation, differentiation, apoptosis, and transformation [34]. In terms of cancer progression, TGFβ1 induces EMT through activation of cell motility and invasion [8]. TGFβ1-induced EMT was performed to confirm the function of NCKAP1 in inhibiting EMT. Figure 3A shows that the cellular morphology was changed to a mesenchymal and fibrotic shape by TGFβ1 treatment in HCT116 colon cancer cells. On the other hand, NCKAP1 knockdown cells showed no change caused by TGFβ1 and still maintained a round and compact shape in the TGFβ1-treated group. The TGFβ1-induced wound healing ability exhibited a very significant difference. The area of wound healing was calculated by measuring the repaired distance and was significantly reduced by downregulation of NCKAP1, even in TGFβ1-treated cells (Fig. 3B-C). CTNNB1 expression was recovered by siNCKAP1 in TGFβ1-treated colon cancer cells (Fig. 3D). NCKAP1 expression did not change in TGFβ1-induced conditions; however, EMT-related genes showed dynamic changes that mesenchymal markers were induced by TGFβ1, and the induction failed after NCKAP1 gene knockdown (Fig. 3E). These data suggest that the phenomenon of EMT blockade by NCKAP1 knockdown, even in TGFβ1-induced conditions, results from the induction of adherent proteins.

### Anti-metastasis effect of NCKAP1 gene knockdown in an animal model

**Fig. 4:**
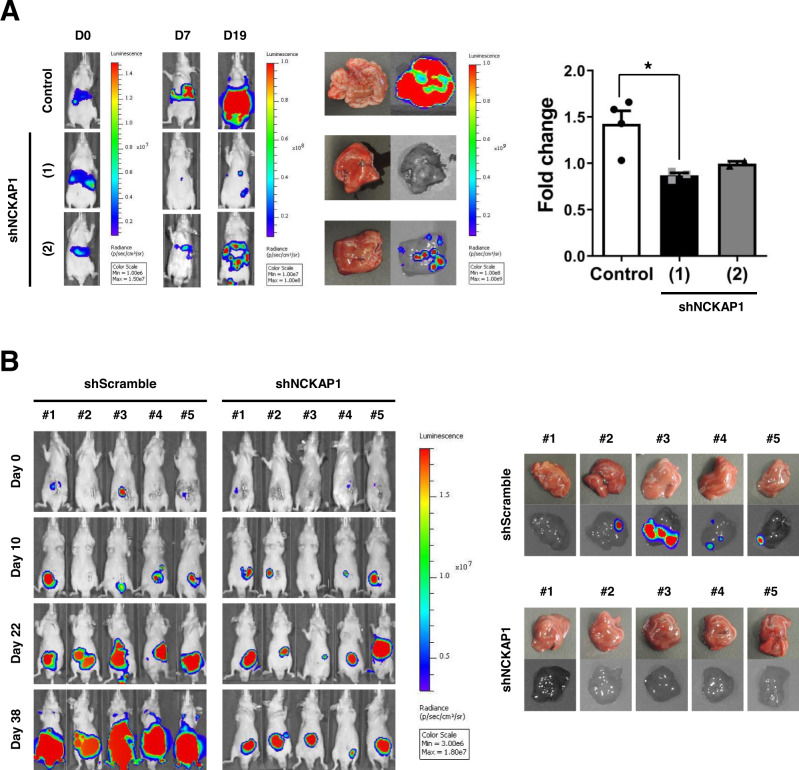
Blockade of metastasis by NCKAP1 knockdown in animal models. **A** A liver metastasis animal model was developed via intrasplenic injection of shNCKAP1-transfected HCT116-Luc cells (5 × 10^6^ cells/50 μl), and splenectomy was performed after 5 min of circulation in BALB/c nude mice. Images of metastatic tumor growth were monitored by the IVIS® Spectrum at 0, 7, and 19 days. Mice with metastatic livers were sacrificed at the endpoint of 22 days. Quantitative results on the right: the graph shows the fold change of liver weight/body weight ratio, and data are reported as the mean ± SEM. One-way ANOVA and Bonferroni’s post hoc test. *p < 0.05 vs. Control. (Control *n* = 4, shNCKAP1 (1) *n* = 3, shNCKAP1 (2) *n* = 2). **B** Tumor tissues for orthotopic transplantation were prepared from shScramble or shNCKAP1-transfected HCT116-Luc cells (5 × 10^6^ cells/50 μl) tumor tissues grown subcutaneously in the BALB/c nude mice. The tumor growth was monitored by for 38 days. Livers were isolated on day 38, and images of liver metastasis were measured by the IVIS® Spectrum.

We established stable cell lines in which NCKAP1 was knocked-down by transfection with a GFP-tagged shRNA construct. NCKAP1 expression was successfully downregulated in shNCKAP1-transfected HCT116 cells (Fig. S5A), in which the wound healing ability was reduced (Fig. S5B). To evaluate the in vivo growth of shNCKAP1-transfected cells, cells were subcutaneously implanted into the hind legs of BALB/c-nu/nu mice, and the growth curve demonstrated no difference between shScramble and shNCKAP1-transfected cells (Fig. S5C). Liver metastases in CRC are a frequent and common problem for cancer therapy. Various animal models have been applied to investigate the mechanisms of cancer. We used two xenograft models of CRC, experimental liver metastasis, and orthotopic xenograft models. In the liver metastasis animal model, colon cancer cells were induced to directly migrate into the liver via splenic injection. Metastatic tumor growth was monitored by the in vivo imaging system (IVIS)® Spectrum for 22 days. Two lines of shNCKAP1-transfected HCT116 cells showed decreased tumor burdens on bioluminescence imaging. On the 22^nd^ day, mice were sacrificed and dissected, and the ratios of liver and whole body weight were measured to analyze metastatic tumor growth. The fold change was calculated as a relative value to the weight ratio of intact mice. As a result, shNCKAP1-transfected cells resulted in inhibition of metastatic tumor growth, especially in shNCKAP1 (1) construct-transfected cells (Fig. 4A). An orthotopic xenograft model was established to observe spontaneous metastasis of colon cancer cells into liver tissue. Tumors were successfully grown in all groups of HCT116 colon cancer cells; however, the growth rate of shScramble-treated cells was more rapid than that of NCKAP1 knockdown cells. In the liver, metastatic tumors were found only in the control group in which the occurrence of metastasis showed an 80% (4/5 mice) rate (Fig. 4B, Fig. S6). These in vivo data demonstrated that NCKAP1 regulates metastasis into distant organs and tumor growth in the mimic microenvironment.

### NCKAP1 as a novel diagnostic marker for CRC

**Fig. 5:**
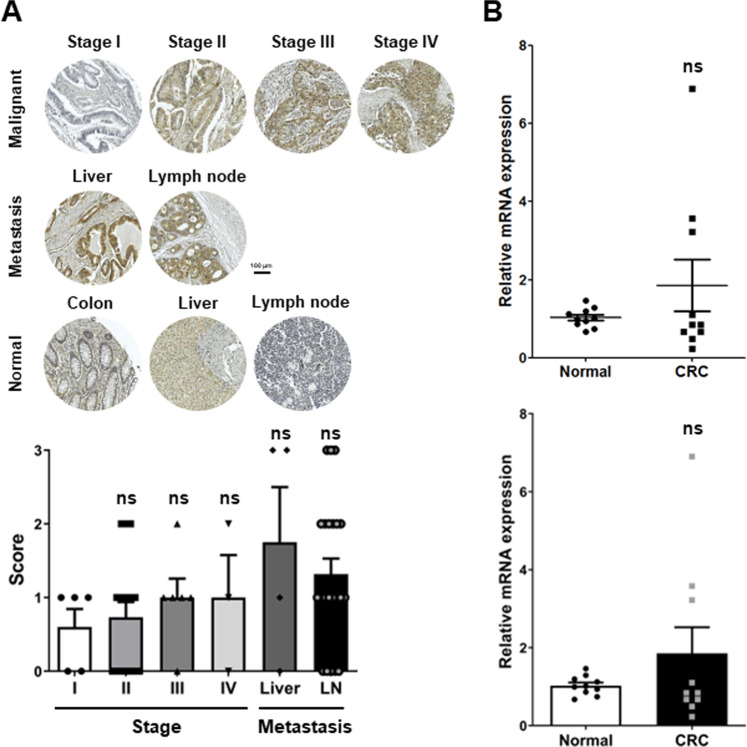
Validation of the possibility of NCKAP1 as a cancer diagnostic marker. **A** IHC was performed with anti-NCKAP1 from TMA. Bar, 100 μm. Quantitative results on the right: the graph represents the score of NCKAP1. Data are reported as the mean ± SEM. One-way ANOVA and Bonferroni’s *post hoc* test. ns *p* > 0.05 vs. Stage I. (Stage I: *n* = 5, II: *n* = 16, III: *n* = 6, IV:3, Liver: *n* = 4, LN: *n* = 22). **B** The mRNA expression of NCKAP1 was confirmed by qRT-PCR analysis in blood buffy coats from patients with colon cancer. The bar graph below the dot graph represents the mean. Data are reported as the mean ± SEM. Student’s t-test. ns: non-significant (*n* = 10).

As a novel diagnostic marker, the feasibility of NCKAP1 was assessed in patient samples of tumor tissues and bloods. The expression of NCKAP1 was analyzed on a global TMA, including malignant primary tumors, metastatic and normal tissues. NCKAP1 levels were scored from 0 to 2, and the scores were classified by the stage of cancer (Fig. 5A). The expression of NCKAP1 was weakly detected in normal colon epithelium and liver; nevertheless, it showed a positive correlation with the cancer stage. Metastatic tumors in the liver most strongly manifested NCKAP1, which indicates that NCKAP1 can represent the prognosis of the disease. The blood level of NCKAP1 was determined by quantitative PCR analysis in buffy coats stored via liquid biopsy. We used 10 samples from healthy donors and patients with CRC for the analysis. In patients with CRC, the NCKAP1 levels were broadly expanded, and the high expression group had higher sensitivity of detection than normal samples (Fig. 5B). These results suggested that NCKAP1 might be applied as a novel marker for diagnosing the CRC stage and determining the prognosis of cancer progression.

## Discussion

Biomarkers are becoming a major means of advancing personalized medicine and are divided into nucleic acid-based biomarkers based on DNA and RNA and protein-based biomarkers based on proteins and parts of them. These biomarkers have been recently applied for early diagnosis of various incurable diseases such as cancer [35] and for drug response and treatment monitoring [36, 37]. Most CRC does not have early symptoms and requires regular diagnostic tests. A blood test is a noninvasive method that is very simple and repeatable. This liquid biopsy is expected to be a useful cancer diagnostic technique for diagnosis, evaluation, and monitoring of treatment efficacy [37–39]. In the clinic, the level of carcinoembryonic antigen (CEA) in the blood is used for diagnosis and indicates the prognosis of CRC [40–44]. However, the correlation between the blood CEA concentration and prognosis remains low because these levels may be increased in patients with cirrhosis, liver disease, and lung cancer and in smokers [45–50]. To overcome these limitations, novel CRC diagnostic markers are needed, and biological characterization studies for that are also needed.

This study revealed that NCKAP1 plays a role as a novel biomarker for diagnosing CRC or predicting the prognosis of CRC metastasis. Specifically, novel CTC target candidates of CRC were discovered through a large-scale gene expression analysis in the blood of a PDX mouse model (Fig. S1 and Fig. S2), and NCKAP1 is one of them that was overexpressed in the blood and tissues of CRC PDX models (Fig. 1). To determine the correlation between NCKAP1 and CRC metastasis, we observed that cancer cell migration and invasion were inhibited by suppressing the expression of NCKAP1 in CRC cell lines (Fig. S3 and Fig. 2A-D). When NCKAP1 expression was decreased by siRNA transfection in colon cancer cells, the levels of CDH1 and CTNNB1, which are known as epithelial marker among EMT markers, were increased (Fig. 2E and Fig. S4A). F-actin expression was also increased in siNCKAP1-treated colon cancer cells (Fig. S4B). NCKAP1 is one of the components of the Wiskott–Aldrich syndrome protein family member (WASF, known as WAVE) regulatory complex (WRC) and binds with remaining four units (Abelson interactor 1 (ABI1), cytoplasmic FMR1 interacting protein 1 (CYFIP1), WASF1, hematopoietic stem/progenitor cell protein 300 (HSPC300)) [51]. In the Rac1 activation condition, WRC is split into the CYFIP1/NCKAP1/ABI1 subcomplex and WASF1/HSPC300 subcomplex [51]. The CYFIP1/NCKAP1/ABI1 subcomplex interacts with Rac1. The WAVE complex actives via Rac signaling, and consequentially, the WASF1/HSPC300 subcomplex interacts with actin-related protein 2/3 complex (Arp2/3); then, actin monomers are converted to actin filaments [25, 31, 51, 52]. It is reported that upregulation of WASF1 induces downregulation of E-cadherin [53]. The suppression of NCKAP1 expression blocks activation of the Rac1-dependent WASF complex [25]. NCKAP1 is critically involved in cytoskeletal remodeling and metastasis [54]. Therefore, the interaction of NCKAP1 and WRC provides a potential target for metastasis mechanisms. When NCKAP1 expression was inhibited by siRNA treatment, actin organization was destroyed, and even actin assembly was not restored by WAVE overexpression in NCKAP1 knockdown cells [54]. Collectively, we suggest that low NCKAP1 induces actin polymerization and EMT inhibition, may related to the Rac-WRC-Arp2/3 pathway. We examined whether NCKAP1 knockdown has significance for metastasis inhibition in TGFβ1-induced EMT situation. TGFβ1-treated colon cancer cells underwent morphological changes, including adopting a mesenchymal shape, but NCKAP1 knockdown cells did not change to a mesenchymal morphology after treatment with TGFβ1 (Fig. 3A). Interestingly, while the wound gap was more rapidly closed in TGFβ1-treated colon cancer cells, the gap area closure caused by treatment with TGFβ1 was inhibited by NCKAP1 knockdown (Fig. 3B-C). β-catenin regulates cell adhesion through interaction with E-cadherin [55]. Epithelial character was weakened in TGFβ1-treated colon cancer cells, but their epithelial properties did not disappear in the NCKAP1 knockdown state even when they were treated with TGFβ1 (Fig. 3D). The mRNA levels of mesenchymal markers (FN, SLUG, CDH2) were increased by TGFβ1 treatment. However, NCKAP1 expression was not affected by TGFβ1 treatment. Furthermore, the increase in FN induced by TGFβ1 was decreased in NCKAP1 knockdown colon cancer cells (Fig. 3E). Taken together, NCKAP1 plays an important role in inhibition of TGFβ1-mediated EMT.

In the two liver metastasis models using shNCKAP1-stable colon cancer cell lines, liver metastasis was suppressed in orthotopic metastasis model as well as in the experimental metastasis model (Fig. 4 and Fig. S6). We experimentally verified the potential of NCKAP1 as a novel CRC target in clinic. We investigated the expression of NCKAP1 using tumor tissues and blood from patient samples. Unlike the data results with high expression of NCKAP1 in samples from patients with CRC, statistical significance was not demonstrated (Fig. 5). We suggest that the number of samples was too small to have a significant *p-value*. It is very unfortunate that we did not find significance, but in most cancer patient monitoring cancer can be accurately diagnosed through imaging and biopsy after cancer-specific markers are detected in blood tests. There is hardly a 100% accurate method that can express metastatic cancer at the time of initial diagnosis. Therefore, we believe that our results are meaningful just by suggesting new possibilities. Although the further experiment could not proceed because the IRB approval period was over, we are confident that significant results will come out if we obtain a larger number of samples. Although NCKAP1 was detected in buffy coat in this study (Fig. 5B), we believe that further developing a more sensitive method to detect NCKAP1 in small amounts of blood would help rapid clinical application, and which is what we need to do in further study. Indeed, since results differ depending on the separation method in the existing separation ability verification method [56, 57], the development of a method for detecting cancer-specific markers even in a small amount of blood is being developed worldwide [17, 21]. In our point of view, this study is meaningful enough to suggest a novel monitoring marker for metastasis that can be detected in blood. Prognostic evaluation of individual patients has very important clinical significance, and cancer diagnosis and prognosis can be predicted through the isolation of cancer cells in the blood and through molecular biological analysis. By discovering cancer cell-specific biomarkers in the blood, additional information on the metastasis of malignant tumors can be provided, and it can be used to select patient-specific therapeutic agents and develop new drugs to overcome resistance in the treatment of metastatic or recurrent cancer.

In summary, we identified that NCKAP1 can be a novel CTC diagnostic marker for CRC. Moreover, a low level of NCKAP1 induces upregulation of CDH1 and CTNNB1 expression, polymerization of actin, and inhibition of metastasis, including migration and invasion even in TGFβ1-mediated mesenchymal conditions. Two kinds of liver metastasis mouse models using NCKAP1 knockdown stable colon cancer cell lines showed dramatic inhibition of tumor growth and metastasis. Although there was no significance, NCKAP1 expression was increased in the tumor comparing with in the normal samples. Therefore, it is meaningful enough to discover and verify a new target in a metastatic colorectal cancer model and observe the phenomenon of inhibiting metastasis. this study emphasizes that colon cancer diagnosis using the expression level of NCKAP1 and the development of NCKAP1 gene expression inhibitors can be useful treatment strategies in the clinic.

## Materials and methods

### Animal models

The animal study was approved by the Institutional Animal Care and Use Committee (IACUC) of the Asan Institute for Life Science (2013-12-156), the Institutional Review Board (IRB) of Asan Medical Center (2010–0618, 2015-0522).

BALB/c nude mice (6-weeks-old male, SLC, Shizuoka, Japan) were used to generate mouse models. Mice anesthetized by an *i.p*. injection of Zoletil (40 mg/kg) plus Rompun (10 mg/kg) mixture were subcutaneously (*s.c*.) transplanted with a piece of cancer tissue into the flank [58]. When the tumor volume reached 250, 500, and 1000 mm^3^, whole blood samples were collected in EDTA tubes through cardiac puncture under anesthesia. Mice were subsequently sacrificed, and then tumor tissues were dissected for weighing and paraffin embedding.

CRC metastasis models were generated using, colon cancer cell lines by orthotopic or experimental implantation. For orthotopic models, a chapped piece of xenograft model was orthotopically sub-transplanted using the cecal pouch method. An experimental metastasis model was developed via intrasplenic injection of colon cancer cells (5 × 10^6^ cells/50 μl), and splenectomy was performed after 5 min of circulation [59]. Tumor growth was monitored through the IVIS spectrum (Perkin Elmer, Massachusetts, USA), and optical images of the whole body were obtained during tumor growth.

### Cell culture

HCT116-Luc and HT29-Luc (ATCC, Virginia, USA) cells were maintained in RPMI1640 media (Invitrogen Gibco, New York, USA) supplemented with 10% (v/v) fetal bovine serum (Invitrogen Gibco, New York, USA) and 100 units/ml penicillin and 100 μg/ml streptomycin (Invitrogen Gibco, New York, USA). For functional study, Smart pool siNCKAP1 and Smart pool siControl were purchased from Dharmacon (GE Healthcare Life Science, Massachusetts, USA). Cells were transfected with 20 nM siRNA by Lipofectamine® RNAiMAX Reagent (Invitrogen, Massachusetts, USA). To establish stable cell lines, shNCKAP1 constructs containing four gene-specific shRNA expression vectors in pGFP-C-shLenti plasmid (OriGene, Maryland, USA) were transfected with Lipofectamine® 2000 Reagent (Invitrogen, Massachusetts, USA). Transfected cells were selected by FACS (BD Biosciences, California, USA) using their GFP expression, and a GFP-positive single cell was cultivated for in vivo analysis.

### TGFβ1-induced EMT

HCT116-Luc cells were transfected with siRNA for 48 h and then the serum starvation (1% FBS + 1% P/S in RPMI) was performed for 24 h. After starvation, siRNA transfected HCT116-Luc cells were treated with 5 ng/ml recombinant human TGFβ1(R&D, Minnesota, USA) for two days.

### Wound healing assay

HCT116-Luc cells were transfected with 20 nM siNCKAP1 and 20 nM non-targeting siRNA (siControl) for 24 h. Transfected HCT116-Luc cells were seeded (1 × 10^5^ cells/50 μl/well) on Culture-Insert 2 Well in a 35-mm µ-Dish (ibidi, Gräfelfing, Germany). HCT116-Luc-shNS or -shNCKAP1 cells were seeded (1 × 10^5^ cells/50 μl/well) on Culture-Insert 2 Well in 35-mm µ-Dish. After cell attachment for 24 h, the insert was removed using sterile tweezers to perform a scratch wound. Cell migration was monitored for 24 h and visualized by an IX71 microscope (Olympus, Tokyo, Japan).

### Migration and invasion assay

For the invasion assay, 8-μm pore Transwell inserts (Corning, New York, USA) were coated with 150 μg/100 μl Matrigel (BD Biosciences, California, USA) for 30 min. After coating with Matrigel suction, siControl or siNCKAP1 transfected HCT116-Luc Cells were seeded (1 × 10^4^ cells/100 μl) on Matrigel-coated Transwell inserts in FBS-free media. Plate wells were filled with 10% FBS media (650 μl/well). The migration assay was performed in the same protocol as in the invasion assay without Matrigel (5-μm pore Transwell inserts (Corning, New York, USA)). After incubation for 48 h at 37 °C, the cells on the Transwell insert were removed with a cotton swab, washed with PBS, stained with 0.5% crystal violet for 30 min, and washed with DW. Images were obtained from BX53 microscope (Olympus, Tokyo, Japan).

### RNA extraction and microarray analysis

Total RNA was extracted from the uterine tissues using the RNeasy mini kit (Qiagen, Maryland, USA). RNA was pooled from the uteri of three mice per genotype and treatment. All RNA samples were analyzed with a Bioanalyzer 2100 (Agilent Technologies, California, USA) before microarray hybridization. Microarray data analysis was performed as previously described. To adjust arrays to a common baseline using invariant set normalization, DChip Analyzer dChip was used, and the perfect match (PM) model described by Li and Wong [60] was used to estimate expression. We selected differentially expressed genes within each treatment in Mig-6f/f and Mig-6d/d mice using a two-sample comparison according to the following criteria: lower boundary of 90% confidence interval of fold change greater than 1.2 and an absolute value of the difference between group means greater than 80. DEGs were classified according to the canonical pathway analyzed by Ingenuity System Software (Ingenuity Systems Inc., California, USA).

### Quantitative real-time PCR analysis

Total RNA was extracted from the uterine tissues using the RNeasy total RNA isolation kit (Qiagen, Maryland, USA). The expression levels of mRNA were measured by real-time PCR TaqMan analysis using an Applied Biosystems StepOnePlusTM system (Applied Biosystems, Massachusetts, USA) and real-time PCR SYBR Green detection system (Bio-Rad, California, USA) according to the manufacturer’s instructions. mRNA quantities were normalized against the housekeeping gene, 18 S RNA. Primer sequences used in these studies are shown in Table S1.

### Western blotting

Cells were washed in PBS and lysed in 2x SDS sample buffer (ELPIS-BIOTECH, Daejeon, South Korea). Membranes were incubated for overnight with anti-β-actin (SIGMA, Massachusetts, USA), anti-α-tubulin (Cell Signaling Technology, Inc., Massachusetts, USA), anti-NCKAP1(Abcam, Cambridge, UK), at 4 °C. And membranes were incubated further for 1 h at room temperature with peroxidase-conjugated donkey anti-rabbit (Jackson ImmunoResearch Laboratories, Inc., Pennsylvania, USA), or donkey anti-mouse antibodies (Jackson ImmunoResearch Laboratories, Inc., Pennsylvania, USA). Detection of protein bands was performed using ECL (Amersham Life Science, Buckinghamshire, UK) and ImageQuant LAS-4000 (GE Healthcare Life Sciences, Massachusetts, USA).

### Immunostaining

HCT116-Luc cells and HT29-Luc cells were seeded (1 × 10^5^ cells/well) on 10-mm round cover glasses in 6-well plates. After 48 h of transfection with 20 nM siControl or siNCKAP1, the slide glasses were washed with PBS (pH 7.5) and fixed in 4% (w/v) paraformaldehyde. The fixed cells were permeabilized with 1:40 diluted Triton X-100 solution and blocked with 0.5% BSA in PBST and incubated with anti-a tubulin (Cell Signaling Technology, Inc., Massachusetts, USA) for 2 h at 37 °C and anti-CDH1(atlas antibodies, Zurich, Switzerland), anti-phalloidin (Invitrogen, Massachusetts, USA), anti-CTNNB1 (Cell Signaling Technology, Inc., Massachusetts, USA), and anti-NCKAP1 (Abcam, Cambridge, UK), antibodies overnight at 4 °C. Alexa Fluor® 488 donkey anti-rabbit, Alexa Fluor® 594 donkey anti-mouse secondary antibody (Jackson ImmunoResearch Laboratories, Inc., Pennsylvania, USA) were applied for 1 h at 37 °C. Vectashield mounting media with 4′,6-diamidino-2-phenylindole (DAPI; Vector Laboratories, California, USA) was used for mounting and counterstaining. Images were viewed with a IX71 microscope (Olympus, Tokyo, Japan).

Immunohistochemistry analysis was performed as previously described [61]. Uterine sections from paraffin-embedded tissue were incubated with anti-NCKAP1 (SIGMA, Massachusetts, USA) antibody in 10% normal goat serum in PBS overnight at 4 °C. Sections were washed in PBS and incubated with a secondary antibody (Vector Laboratories, California, USA) for 1 h at room temperature. Immunoreactivity was detected using the Vectastain Elite DAB kit (Vector Laboratories, California, USA) and analyzed with a BX53 microscope (Olympus, Tokyo, Japan). The colon cancer tissue microarray (TMA) panel was purchased from US Biomax, Inc (Maryland, USA). The TMA staining was conducted with anti-NCKAP1(SIGMA, Massachusetts, USA) overnight at 4 °C and with antirabbit second antibody (Vector Laboratories, California, USA) for 2 h at room temperature. The scoring of NCKAP1 expression level was performed at the pathology department of Asan Medical Center, Seoul.

### Statistical analysis

All data are expressed as the mean ± SEM to represent at least three different experiments. Statistical analysis was performed by Student’s t-test or one-way analysis of variance using Prism statistical software (GraphPad Software, California, USA). P-values less than 0.05 were considered statistically significant.

For data with two groups, Student’s t test was used. For data containing more than two groups, one-way ANOVA or two-way ANOVA was used, followed by Tukey’s post hoc or Bonferroni’s post hoc multiple range. All data are presented as the mean ± SEM. *p* < 0.05 was considered statistically significant. All statistical analyses were performed using the Instat package from GraphPad (California, USA).

## Supplementary information

Supplemental figures and original data

## Data Availability

All data involved in this study are available in the main text or the supplementary materials.

## Material availability

Materials can be available through agreement with corresponding authors
