# Supplementary material for: NCK-associated protein 1 regulates metastasis and is a novel prognostic marker for colorectal cancer
Source: Cell Death Discov. 2023 Jan 13;9:7. doi: 10.1038/s41420-023-01303-6 (PMC9839720; doi:10.1038/s41420-023-01303-6)

**NCK-associated protein 1 regulates metastasis and is a novel prognostic marker for colorectal cancer**

Mi Ri Kwon^1,2†^, Jae Hee Lee^2†‡^, Jin Park ^2,3^, Seok Soon Park^2,3^, Eun Jin Ju^2,3^, Eun Jung Ko^2,3^, Seol Hwa Shin^2,3^, Ga Won Son^1,2^, Hye Won Lee^1,2^, Yeon Joo Kim^4^, Si Yeol Song^3,4^, Seong-Yun Jeong^2,3,5^*, Eun Kyung Choi^3,4^*.

^1^Department of Medical Science, Asan Medical Institute of Convergence Science and Technology, Asan Medical Center, University of Ulsan College of Medicine, Seoul, 05505, Republic of Korea

^2^Asan Institute for Life Sciences, ASAN Medical Center, Seoul, 05505, Republic of Korea

^3^Asan Preclinical Evaluation Center for Cancer Therapeutix, ASAN Medical Center, Seoul, 05505, Republic of Korea

^4^Department of Radiation Oncology, ASAN Medical Center, University of Ulsan College of Medicine, Seoul, 05505, Republic of Korea

^5^Department of Convergence Medicine, ASAN Medical Center, University of Ulsan College of Medicine, Seoul, 05505, Republic of Korea

**Supplementary Figures S1 to S6**

**Supplementary Table S1**

**Original data files**


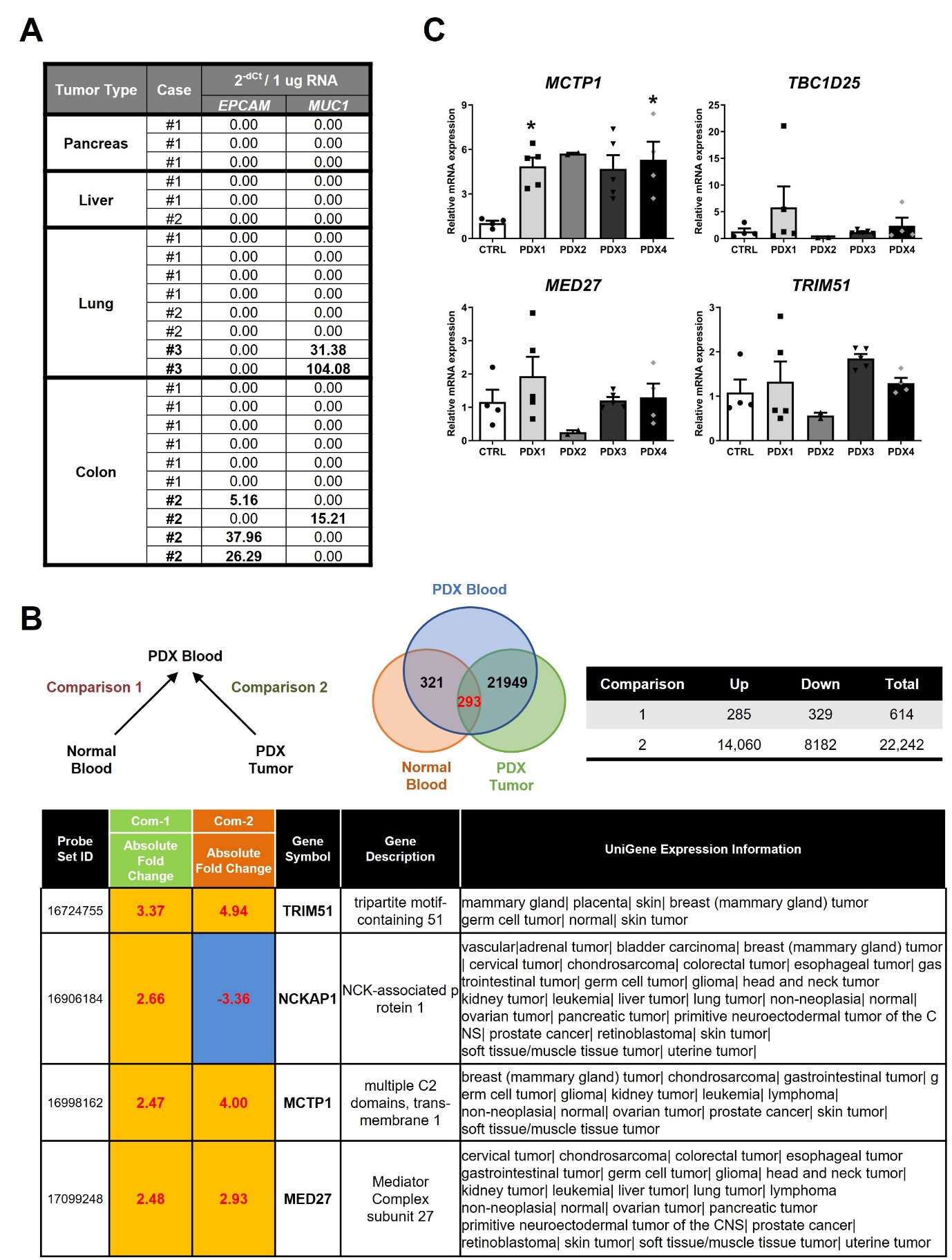


**Supplementary Fig. S1 Gene microarray analysis in the blood cells derived from PDX mice. A** EPCAM and MUC1 gene expression in the blood of human PDX models were quantified by qRT-PCR. **B** Validation of gene expression in RNA was analyzed using qRT-PCR. The schematic diagram: DEG analysis in two comparisons (Com-1: Normal blood vs. Colon cancer PDX blood, Com-2: Colon cancer PDX tumor vs. Colon cancer PDX blood). Table: Summary of target genes identified by fold change of DEG analysis of microRNA between two comparisons. **C** The mRNA level of liquid biopsy from four colon cancer PDX mice was quantified by qRT-PCR and normalized to the expression level of GAPDH. Statistical significance was determined using one-way ANOVA and Tukey’s *post hoc* test (The means ± SEM)**.** **p* < 0.05 vs. CTRL. (CTRL: n= 4, PDX1: n=5, PDX2: n=2, PDX3: n=5, PDX4: n=4).


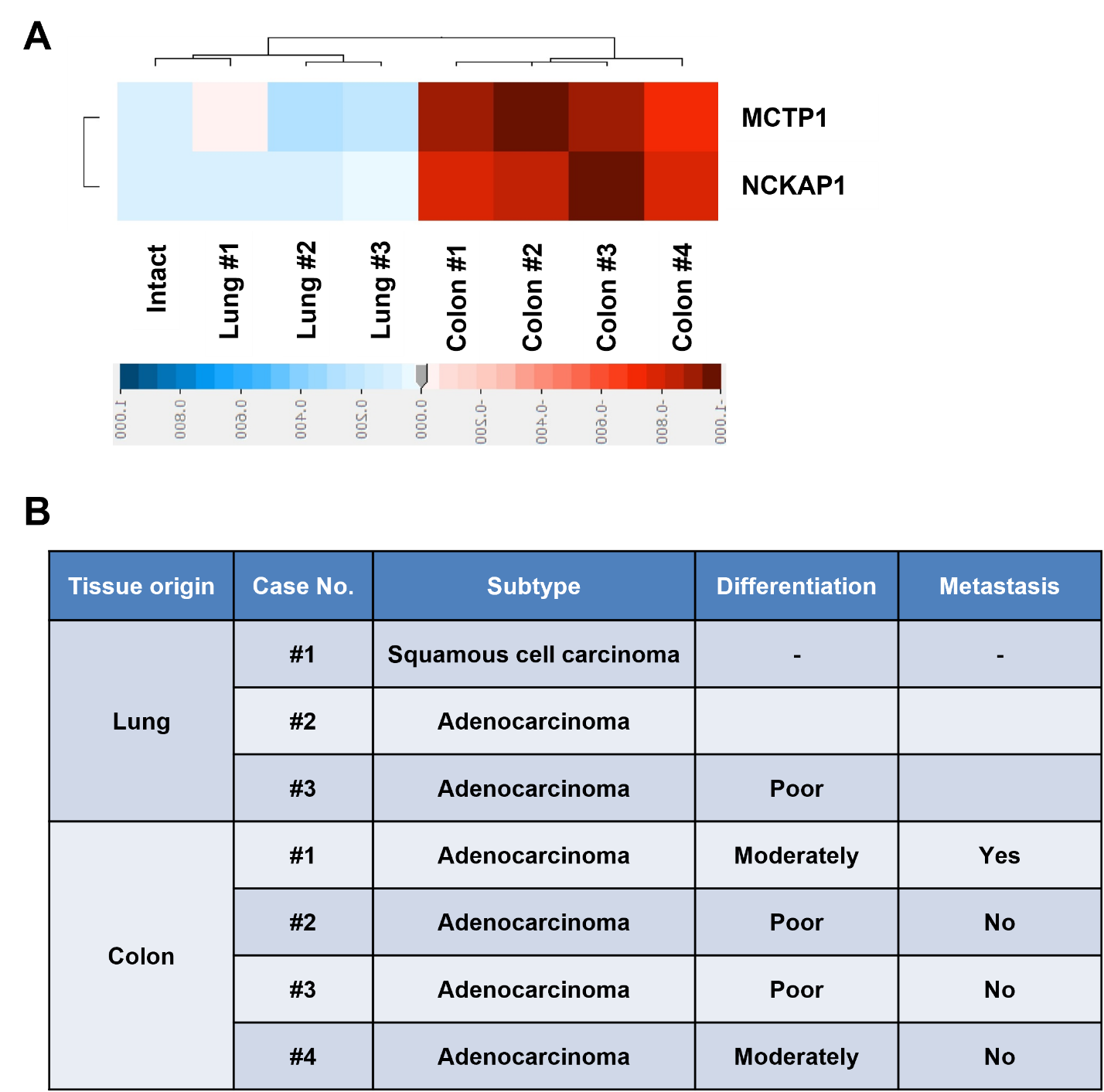


**Supplementary Fig. S2 Gene expression analysis in PDX models. A** A gene heatmap in colon and lung cancer PDX shows the pattern of gene expressions of MCTP1 or NCKAP1 compared with the intact sample. Red indicates high gene expression, and blue indicates decreased gene expression. **B** The table summarizes information on PDX samples used for gene expression analysis.


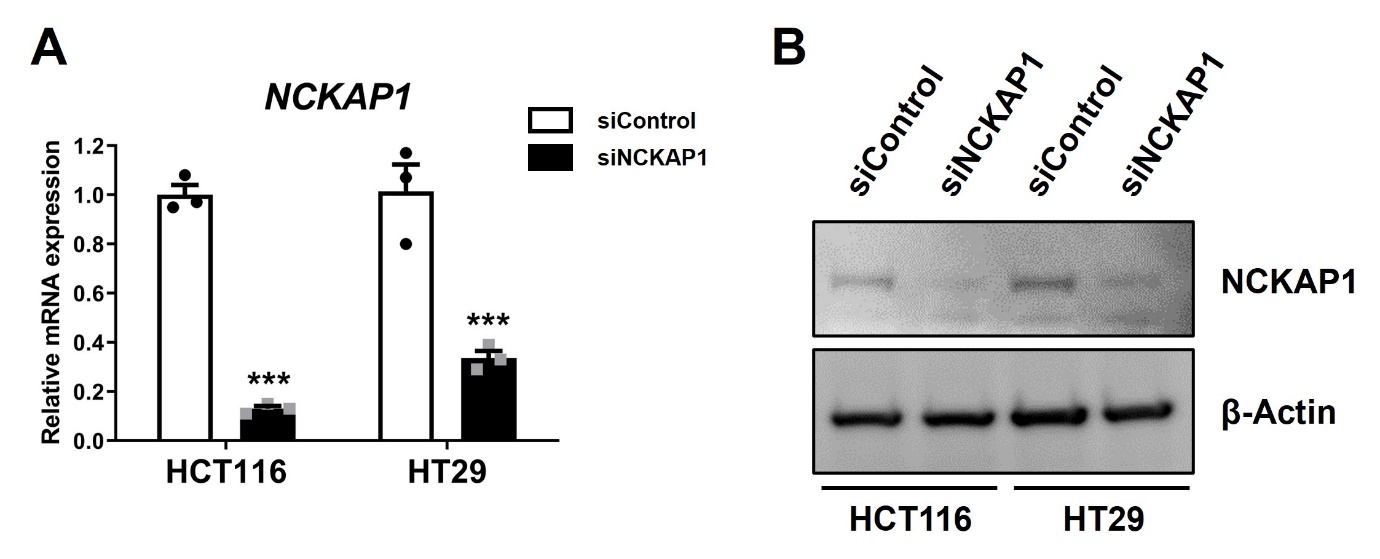


**Supplementary Fig. S3 Confirmation of NCKAP1 knockdown in siRNA-transfected colon cancer cell lines. A** The mRNA level of NCKAP1 was quantified by qRT-PCR. HCT116-Luc cells and HT29-Luc cells were transfected with 20 nM siControl or siNCKAP1 for 72 h. Statistical significance was determined using two-way ANOVA and Bonferroni’s *post hoc* test (mean ± SEM). ****p* < 0.001 vs. siControl. (n=3). **B** NCKAP1 knockdown was confirmed by western blotting. Proteins were prepared from the cells transfected with 20 nM siControl or siNCKAP1 for 72 h, and Western blotting was performed by using anti-NCKAP1 and anti-β-actin.


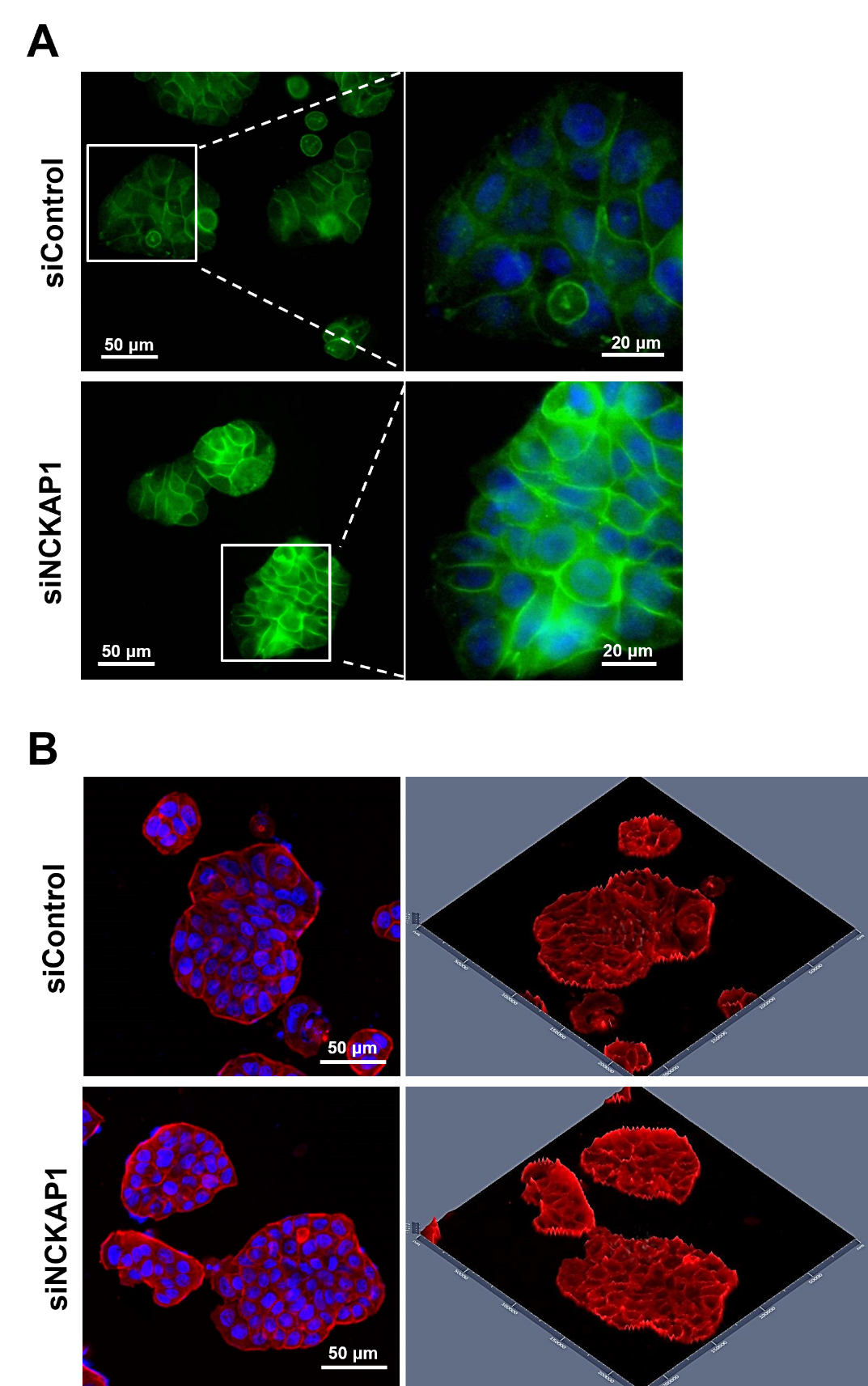


**Supplementary Fig. S4 Identification of the mechanism of NCKAP1 on CRC metastasis. A** HT-29-Luc cells were transfected with 20 nM siControl or siNCKAP1 for 48 h. Immunocytochemistry of CTNNB1 (β-catenin) and DAPI was performed and observed under a confocal microscope. Bar, 20 μm and 50 μm. **B** Transfected HT-29-Luc cells were stained with anti-Phalloidin (F-actin) and DAPI. Representative pictures of cells are shown by confocal microscopy and 3D images. Bar, 50 μm.


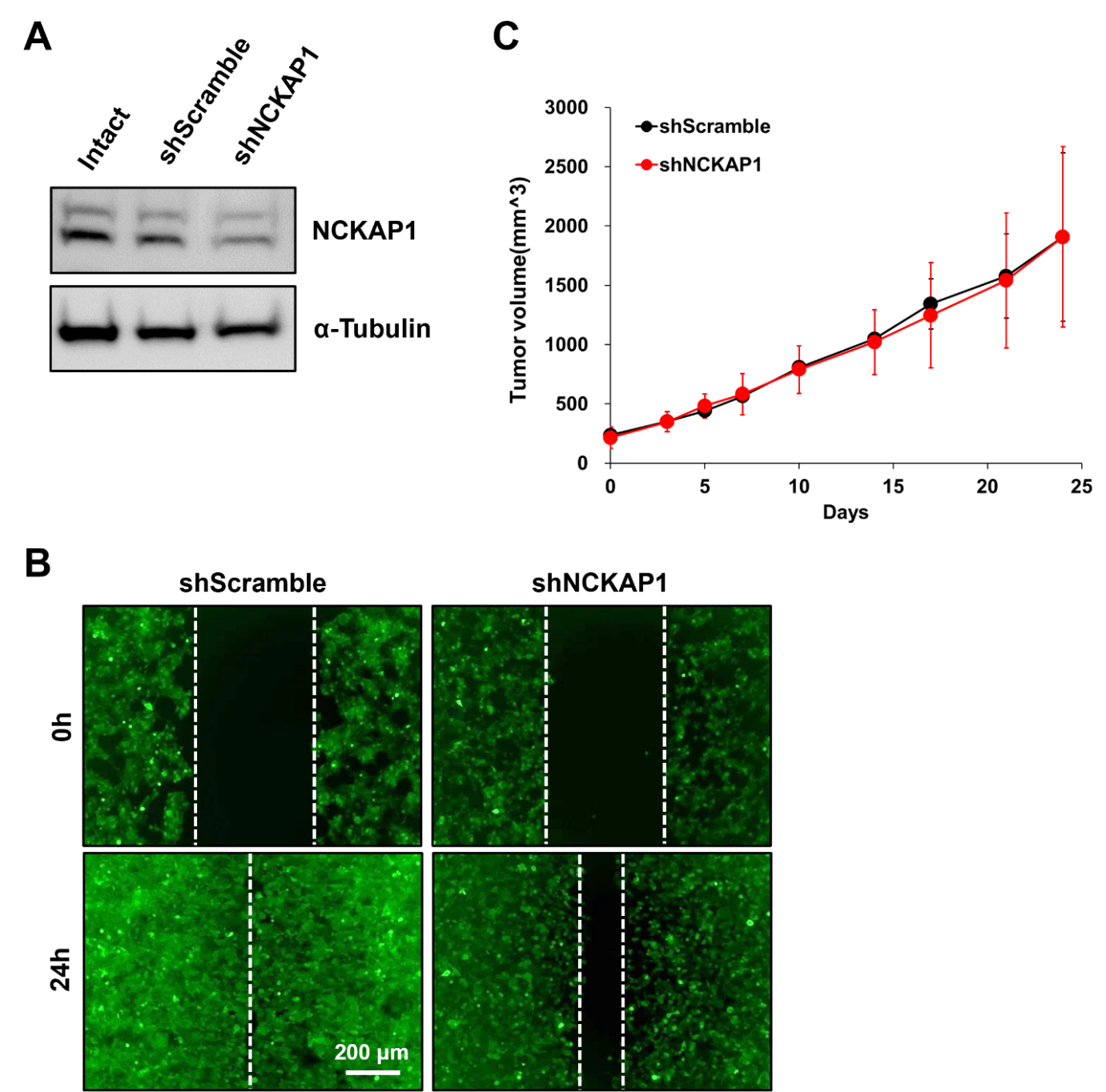


**Supplementary Fig. S5 Confirmation of NCKAP1 knockdown and tumorigenesis in shRNA-transfected stable colon cancer cell lines. A** Proteins were prepared from the shScramble or shNCKAP1-transfected HCT116-Luc stable cells, and NCKAP1 knockdown was confirmed by western blotting of anti-NCKAP1 and anti-α-tubulin. **B** A wound healing assay was performed in shScramble or shNCKAP1-transfected HCT116-Luc stable cells and observed under a confocal microscope for 24 h. Bar, 200 μm. **C** A BALB/c nude mouse was subcutaneously implanted with shRNA-transfected HCT116-Luc stable cells (5x10^6^ cells/50 μl). When the tumor reached 200 mm^3^, tumor sizes were measured every 2–3 days and plotted for growth curve. Data are reported as the mean ± SD. (n=4).


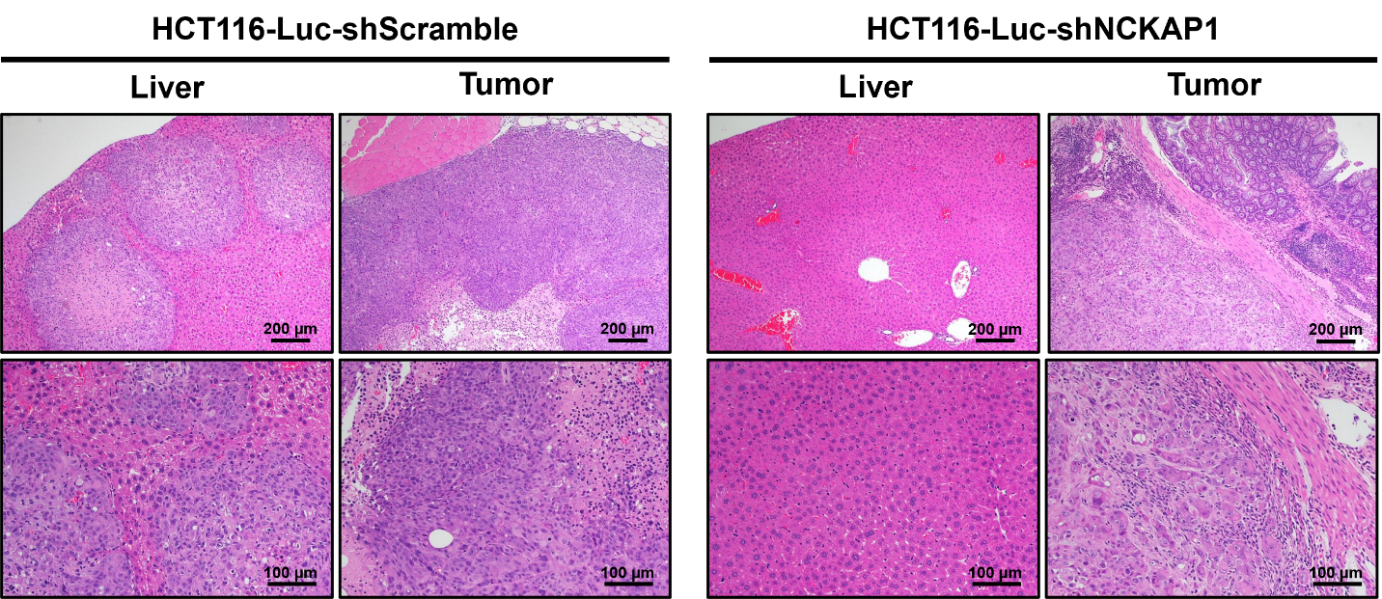


**Supplementary Fig. S6 Histological analysis of metastatic tumors in an orthotopic xenograft model using HCT116-Luc-shRNA stable cells.** Tumor and liver sections were obtained at day 38 in the HCT116-Luc-shRNA orthotopic xenograft model, and H&E (hematoxylin and eosin) staining was performed. Bar, 100 or 200 μm.

**Supplementary Table S1 List of qRT-PCR primer sequences**

| **Primer name** | **Sequence** |
| --- | --- |
| GAPDH-F | AATCCCATCACCATCTTCCA |
| GAPDH-R | TGGACTCCACGACGTACTCA |
| MCTP1-F | TCCCTTCAGATGTACAAGTGGT |
| MCTP1-R | GCTAGCCAAGATTGTTTTTCTTTC |
| TBC1D25-F | CTGAGATTGGGAAGTATGGGCT |
| TBC1D25-R | GCATTGGTGCATAAACCTCACA |
| MED27-F | TACATTGCAACGCAGAGCCTT |
| MED27-R | CCCAGCAAGGAACAAGATGGA |
| TRIM51-F | ATTGCTCCTTCTCACCTCCTC |
| TRIM51-R | GGGCTTTCTGGGATAAACAGGT |
| NCKAP1-F | ACCCGCAACAACAATCAACAG |
| NCKAP1-R | CAGCAATTCACAAACATGGTCC |
| FN (Fibronectin)-F | TAGAAGCCTGCACTTCAAAAGG |
| FN (Fibronectin)-R | GAACAGCTCTGGACTTGGGAT |
| SLUG-F | TGTGTGGACTACCGCTGC |
| SLUG -R | TCCGGAAAGAGGAGAGAGG |
| CDH2-F | GCGCTGGCACCGTTTTTAC |
| CDH2-R | CCTGACCACGAAGAGTGTAGA |

**Figure S3 original western blots**


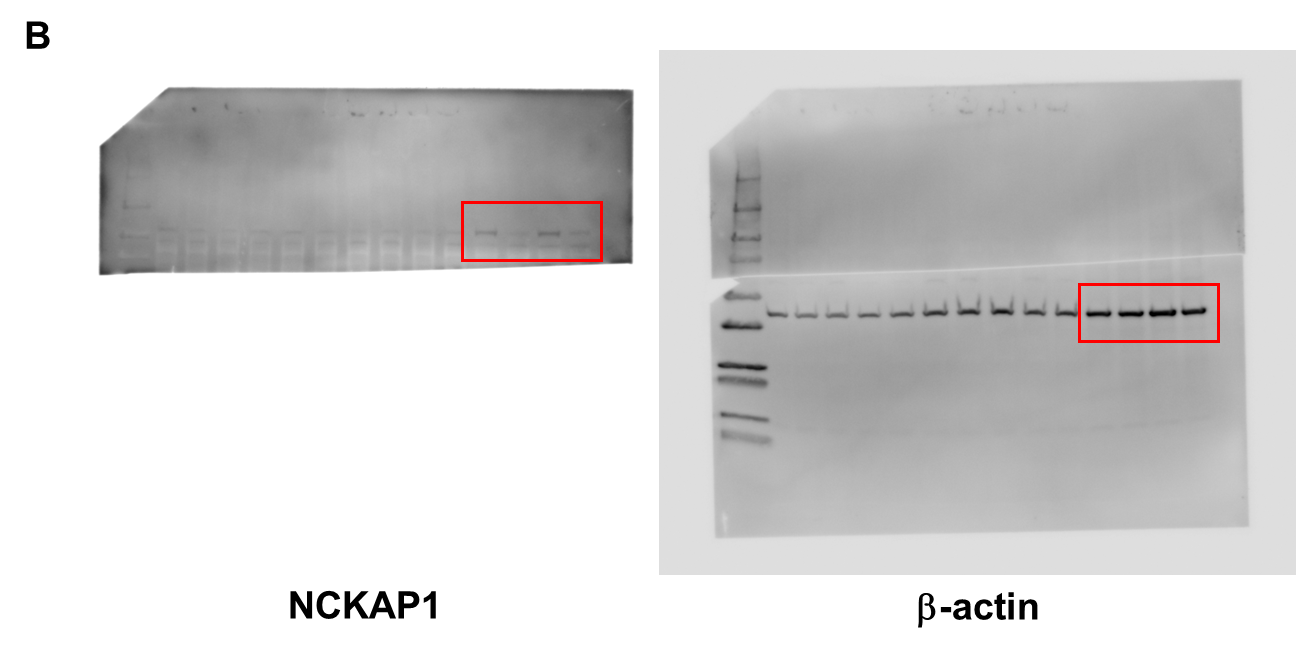


**Figure S5 original western blots**


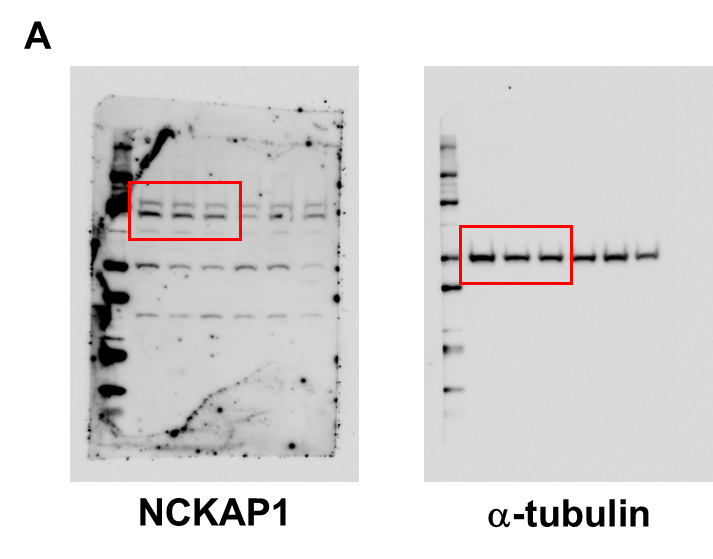

Supplement: Supplementary file 1 — Supplemental figures and original data [file 41420_2023_1303_MOESM1_ESM.docx]
